# Remotely Sensed Environmental Conditions and Malaria Mortality in Three Malaria Endemic Regions in Western Kenya

**DOI:** 10.1371/journal.pone.0154204

**Published:** 2016-04-26

**Authors:** Maquins Odhiambo Sewe, Clas Ahlm, Joacim Rocklöv

**Affiliations:** 1 Centre for Global Health Research, Kenya Medical Research Institute, Kisumu, Kenya; 2 Department of Public Health and Clinical Medicine, Epidemiology and Global Health, Umeå University, Umeå, Sweden; 3 Department of Clinical Microbiology, Infectious Diseases, Umeå University, Umeå, Sweden; Shanxi University, CHINA

## Abstract

**Background:**

Malaria is an important cause of morbidity and mortality in malaria endemic countries. The malaria mosquito vectors depend on environmental conditions, such as temperature and rainfall, for reproduction and survival. To investigate the potential for weather driven early warning systems to prevent disease occurrence, the disease relationship to weather conditions need to be carefully investigated. Where meteorological observations are scarce, satellite derived products provide new opportunities to study the disease patterns depending on remotely sensed variables. In this study, we explored the lagged association of Normalized Difference Vegetation Index (NVDI), day Land Surface Temperature (LST) and precipitation on malaria mortality in three areas in Western Kenya.

**Methodology and Findings:**

The lagged effect of each environmental variable on weekly malaria mortality was modeled using a Distributed Lag Non Linear Modeling approach. For each variable we constructed a natural spline basis with 3 degrees of freedom for both the lag dimension and the variable. Lag periods up to 12 weeks were considered. The effect of day LST varied between the areas with longer lags. In all the three areas, malaria mortality was associated with precipitation. The risk increased with increasing weekly total precipitation above 20 mm and peaking at 80 mm. The NDVI threshold for increased mortality risk was between 0.3 and 0.4 at shorter lags.

**Conclusion:**

This study identified lag patterns and association of remote- sensing environmental factors and malaria mortality in three malaria endemic regions in Western Kenya. Our results show that rainfall has the most consistent predictive pattern to malaria transmission in the endemic study area. Results highlight a potential for development of locally based early warning forecasts that could potentially reduce the disease burden by enabling timely control actions.

## Introduction

Malaria is the most important vector-borne disease in the world contributing to high morbidity and mortality especially in children and pregnant women. Africa bears the greatest burden of the disease accounting for 80% of the 207 million estimated malaria cases reported worldwide in 2012 [1]. In 2012, Kenya was among the 18 countries in Africa that contributed 80% of estimated global malaria deaths [1]. Global concerted efforts to curb the disease in Africa has resulted in reductions on malaria mortality rates by 49% in all ages and 54% in children under five years between the years 2000 and 2012 [1]. A study looking at age-specific malaria mortality rates in two malaria endemic districts in Western Kenya conducted by the Kenya Medical Research Institute and United States Centre for Disease Control (KEMRI/CDC) followed in Health and Demographic Surveillance Systems (HDSS) between the years 2003 and 2010 reported 67% reduction in malaria mortality rates in all ages and 72% reduction in children under five years of age [2]. The massive reductions were attributed to the high prevalence of household bed net ownership of 81%.

Despite these gains in mortality reduction, malaria still contributes to high morbidity and hospital admissions in the region stressing the limited available resources. Control and eradication of malaria is critical for the achievement of Millennium Development Goal four which requires two thirds reduction in child mortality by 2015. The KEMRI/CDC HDSS area lies in the region in Western Kenya that has high malaria parasite prevalence of 38% in children less than 15 years. Malaria with a mortality fraction of 30% is the leading cause of death in children below five years [3]. The region experience continuous, but seasonally varying malaria transmission.

Natural and human modified environments interact to create suitable conditions for high mosquito vector abundance. Malaria incidence increases for households near swampy areas, and where there are agricultural activities such as banana and cacao farming [4, 5]. Similar in Kenya highlands, malaria incidence and larval presence tend to increase in close proximity to swampy areas or streams, and near agricultural lands such as tea plantations [6, 7]. In one study, topological wetness index predicted household malaria risk better than land use [7]. In a lowland region of Western Kenya, it was shown that land cover including mature maize, freshly cultivated lands and newly pasteurized grasslands were associated with *anopheles* larval presence [8].

Rainfall results in pools of water, which provide suitable breeding sites for vectors while temperature determines development of the *anopheles* mosquitoes, adult mosquito biting and mortality rates [9, 10]. In a previous analysis [11], lagged values of weekly mean temperature and total rainfall up to 16 weeks were assessed for their association with malaria mortality among children in KEMRI/CDC HDSS area. Rainfall and temperature data were obtained from Kisumu airport weather station which is approximately 60 km from the study area. This was prompted by unavailability of reliable ground weather data for the study area. Unavailability of weather data at appropriate spatial and temporal scales is experienced in many low-income countries thus limiting estimation of risk of weather and climate-sensitive diseases such as malaria [12]. Therefore in this study, we used satellite derived remote sensing data which is obtained when satellites orbiting the earth picks light or electromagnetic radiations from objects on earth’s surface or from earth itself [13]. For example, NDVI is calculated by using near infrared (NIR) channel and visible light absorbed by vegetation. The index varies from -1 to 1. Negative values indicate water bodies while 1 shows the highest density of green leaves [14]. Remote sensing data has been used in several previous applications in malaria epidemiology and control.

Satellites that have been used in malaria research include Terra with MODIS (Moderate Resolution Imaging Spectroradiometer) sensor and National Oceanic and Atmospheric Administration (NOAA)–M with AVHRR (Advanced very high resolution radiometer) sensor among many more [14, 15].

Data from earth observation satellites has been used in the development of malaria early warning systems [14, 16], development of malaria risk maps [17, 18] and identification of suitable malaria vector habitats [19–21]. Some of the remote sensing data that have been applied to malaria epidemiology include: land surface temperature (LST), cold cloud duration, vegetation indices such as NDVI and enhanced vegetation index, precipitation and actual evapotranspiration [19–21].

The objective of this research was to study the association between remote sensing variables: day LST, precipitation and NDVI, on malaria mortality over time in KEMRI/CDC HDSS areas with a higher resolution to better understand to what extent weather variability is driving the malaria mortality patterns in the regions.

## Materials and Methods

### Ethics Statement

The protocols for KEMRI/CDC HDSS are approved by both CDC (# 3308, Atlanta, GA) and KEMRI (# 1801, Nairobi, Kenya) Institutional Review Boards. The individual level mortality data was anonymized and de-identified through aggregation to weekly level and stripping of individual level identifiers.

### Study area

The KEMRI/CDC HDSS covers three areas in Western Kenya following a population of over 240,000 individuals. The three areas are Asembo in Rarieda district, Gem in Gem district and Karemo in Siaya District. Asembo was the first to be enumerated in 2001, followed by Gem in 2002 and Karemo in 2007. Censuses are conducted every four months where demographic and socio-economic data such as births, deaths, migrations, education and socio-economic status are updated. The KEMRI/CDC HDSS has been described in detail elsewhere [22, 23]. Verbal Autopsies are conducted on all deaths and the signs and symptoms are used to determine probable cause of death.

### Malaria Mortality Data

We used malaria cause of death data for the KEMRI/CDC HDSS for the period 2003 to 2012 for Asembo and Gem areas while 2008 to 2012 for Karemo. Baseline data collection in Karemo began in 2007 [22], thus complete annual time series starts from 2008 in that area. The daily data was aggregated to weekly temporal resolution for each of the three areas and all areas combined.

To obtain malaria deaths Inter-VA4 method was used. This is probabilistic method for deriving probable cause of death from Verbal Autopsy (VA) questionnaire. The VA details signs and symptoms exhibited by the diseased prior to death. These signs and symptoms in binary categories are then fed to the Bayesian probabilistic algorithm to derive the possible cause of death. This is a more consistent method to derive cause of death data from VA questionnaires compared to physician coding previously employed in KEMRI/CDC HDSS [24, 25].

### Remote sensing data

The remotely sensed data obtained included day LST, precipitation and NDVI. The day LST and NDVI were extracted from Terra MODIS MOD11A1 and MYD13Q1 products, respectively hosted and managed by United States Geological Survey and National Aeronautics and Space Administration *(*NASA) [26].

**Fig 1:**
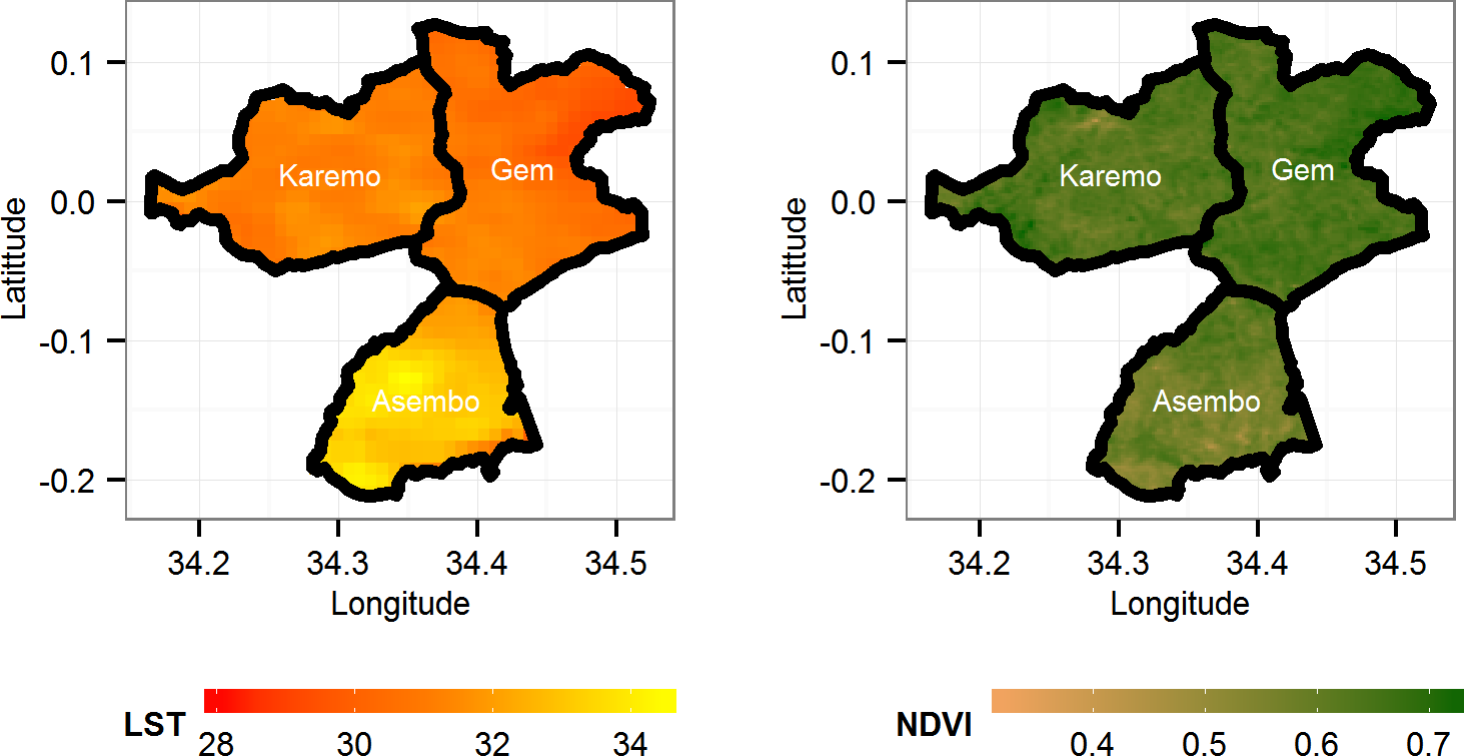
Map showing LST and NDVI for the three study areas in Western Kenya aggregated for the years 2003–2012.

The day LST was available at daily temporal and 1000m spatial resolution, while NDVI data was available at 16 days temporal and 250m spatial resolutions, respectively, as displayed in Fig 1. The MODIS datasets were downloaded as HDF-EOS (Hierarchical Data Format—Earth Observing System) files. We downloaded two tiles (h21v09) and (h21v08) that cover the whole KEMRI/CDC HDSS study area for the period 2003 to 2012. The downloaded HDF image tiles were stitched together and re-projected using MODIS projection tool [26] to TIFF images in the R 3.10 environment. The TIFF images were processed using RGDAL [27] package in R software to extract LST and NDVI for each of the compound in the three HDSS areas and then aggregated per area. The precipitation data was downloaded from NASA’s Tropical Rainfall Measuring Mission (TRMM) as binary files at 0.25° x 0.25° spatial resolution and daily at three-hour intervals temporal resolution. To get total daily precipitation estimates, we multiplied the hourly rates by 3 and summed.

The 16 day MODIS NDVI data was interpolated using natural cubic spline in the TIS package in R to get daily estimates. The missing daily values for LST and precipitation were linearly interpolated using TIS [28] package in R software [29]. The area specific and all areas daily, LST, precipitation and NDVI values were then aggregated to weekly temporal resolution for the study period 2003–2012. For LST and NDVI we computed weekly mean values while for precipitation we computed weekly totals.

### Statistical Analysis

We modelled the delayed effect of day LST, precipitation and NDVI on the weekly malaria mortality using Distributed Lag Non-Linear Models (DLNM) package in R [30]. The DLNM framework allows modelling of non-linear relationships in dimensions of the predictor as well as its lag. The DLNM method employs the concept of cross-basis which is a joint modeling of *basis* functions of the predictor variable and its lag [31]. We created models for each of the study areas, and for all areas together. DLNM for each of the environmental variables was created using a natural cubic spline basis with 3 degrees of freedom to capture the non-linear effects as well as their lag dimensions. We modelled lags 0 to 12 weeks for each of the explanatory variables. Weekly malaria deaths were assumed to follow a quasi-Poisson process allowing for over-dispersion. In each model, a natural cubic spline function of time trend allowing one degree of freedom per year of data was included to capture long-term time trends of malaria mortality based on previous estimation[11]. The model equations used for estimating the effect of each environmental variable on malaria mortality were:

$$ln(E(Y_{t}))=\beta_{o}+s(T,timedf)+f(X_{t},lagdf,vardf)+\beta_{i}X_{i}$$ 1.

$$ln(E(Y_{t}))=\beta_{o}+s(T,timedf)+f(X_{t},lagdf,vardf)+\beta_{i}X_{i}+s(month,vardf)$$ 2.

$$E(Y_{t})∼Poisson$$

where *E*(*Y*_*t*_) is the expected number of malaria deaths in week t. *β*_*o*_ is the intercept, *s*(*T*,*timedf*) is the smooth function of time with degree of freedom *timedf*,*f*(*X*_*t*_,*lagdf*,*vardf*) is the crossbasis function of variable t and its lag dimension with vardf and lagdf degrees of freedom respectively controlling for the the ith covariate *X*_*i*_. For example, the model for precipitation included LST day and NDVI as linear predictors. We also ran similar models adjusting explicitly for within year seasonality by including a smooth function of month with 3 degrees of freedom shown in Eq 2 with the additional s(month,vardf) component. The cross-basis functions of day LST, precipitation and NDVI were centered at 28°C, 20 mm and 0.4 respectively for each of the areas. Centering values were determined by visual examination of the exposure mortality relationships. For NDVI we chose a centering value of 0.4 based on a study in coastal town of Kilifi in Kenya [20], which showed an NDVI threshold of 0.3 to 0.4 for increase in malaria incidence. It should be noted that the choice of centering value does not change the relationship between outcome and exposure. Relative risks presented are in reference to these centering points. We also modelled the delayed effect of precipitation on NDVI and computed cross-correlation coefficients.

We plotted the overall effects of each of the remote sensing variables over the whole lag period up to 12 weeks, and also plotted contour graphs showing both the lag effect at the whole range of predictor variable. The lag effects were estimated as 1°C increase in day LST, 0.1 decrease in NDVI below the centering value and 10 mm increase in rainfall above the reference value.

## Results

**Fig 2:**
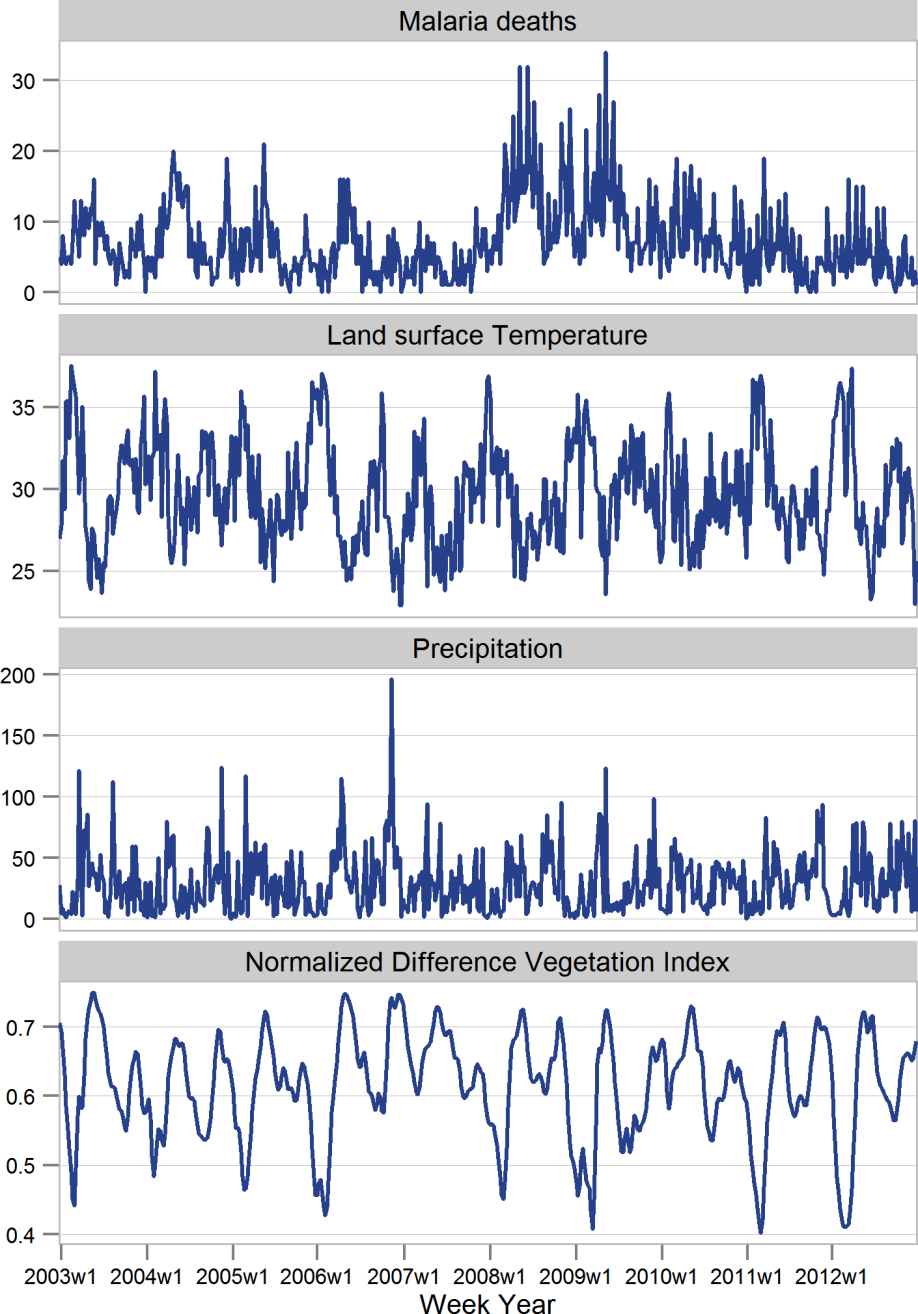
Distribution of weekly mean values of malaria deaths, LST (°C), Precipitation (mm) and NDVI in the areas Asembo, Gem and Karemo, Kenya 2003–2012.

There was a total of 3,809 malaria deaths reported in all the three KEMRI/CDC HDSS areas during the study period. The number of deaths reported in Asembo and Gem was 1,240 and 1,896, respectively in the years 2003 to 2012 while the number of deaths in Karemo was 641 reported between 2008 and 2012. The weekly malaria deaths’ mean was 7.2 in all the three areas combined. Gem had the highest average of 3.6 as displayed in Table 1. There were weeks recording high death counts up to 34 in 2009, the highest peak as shown in Fig 2. The mean day LST varied little between the three areas with a mean of 29.7°C in all areas combined.

**Table 1:** Summary of weekly malaria mortality and remote sensing variables LST (°C), Precipitation (mm) and NDVI, in Asembo, Gem, Karemo and all areas combined.

Variable	Area	Min	Max	Mean	Percentiles				
					5%	25%	50%	75%	95%
Malaria deaths	Asembo	0	13	2.3	0	1	2	3	6
	Gem	0	20	3.6	0	1	3	5	9
	Karemo	0	12	2.4	0	1	2	3	6
	All Areas	0	34	7.2	1	3	6	10	17
Land Surface Temperature	Asembo	22.2	39.1	30.6	25.3	28.0	30.5	33.0	36.2
	Gem	22.5	37.3	29.1	24.5	26.8	28.6	31.3	35.5
	Karemo	19.9	38.6	29.4	24.7	27.0	29.1	31.3	35.4
	All Areas	22.9	37.5	29.7	24.9	27.3	29.5	31.9	35.6
Precipitation	Asembo	0	197.8	27.6	1.8	7.3	22.3	39.5	73.6
	Gem	0	190.9	31.2	3.8	11.6	27.0	42.6	79.6
	Karemo	0	126.3	29.3	1.6	9.5	26.1	41.3	76.5
	All Areas	0	196.2	29.5	2.6	9.5	25.9	41.1	78.0
Normalized Difference Vegetation Index	Asembo	0.34	0.75	0.58	0.43	0.53	0.59	0.65	0.71
	Gem	0.44	0.77	0.64	0.49	0.60	0.65	0.69	0.75
	Karemo	0.40	0.73	0.61	0.45	0.56	0.62	0.67	0.71
	All Areas	0.40	0.75	0.61	0.46	0.57	0.62	0.67	0.72

There was an increase in vegetation index with increasing precipitation and shorter lag effect of precipitation on NDVI for weekly total rainfall over 60mm (S1 Fig). Gem area showed the greatest concentration of green vegetation with the average NDVI of 0.64 and lowest in Asembo at 0.58 (Table 1 and Fig 1).

We observed weekly seasonal patterns in malaria mortality with peaks within each year corresponding to season fluctuations in the remote sensing variables day LST and precipitation (Fig 2). The within year monthly seasonal fluctuation in risk of mortality is depicted by a spline function of month in S4 Fig The highest risk of mortality is observed in April and the lowest risk in September. In 2008, there was an upsurge in malaria deaths, which remained persistent in 2009 and later declined in the year 2010 (Fig 2). Vegetation Index also displayed a within year bimodal seasonal pattern. Precipitation showed yearly variation with two distinct peaks.

**Fig 3:**
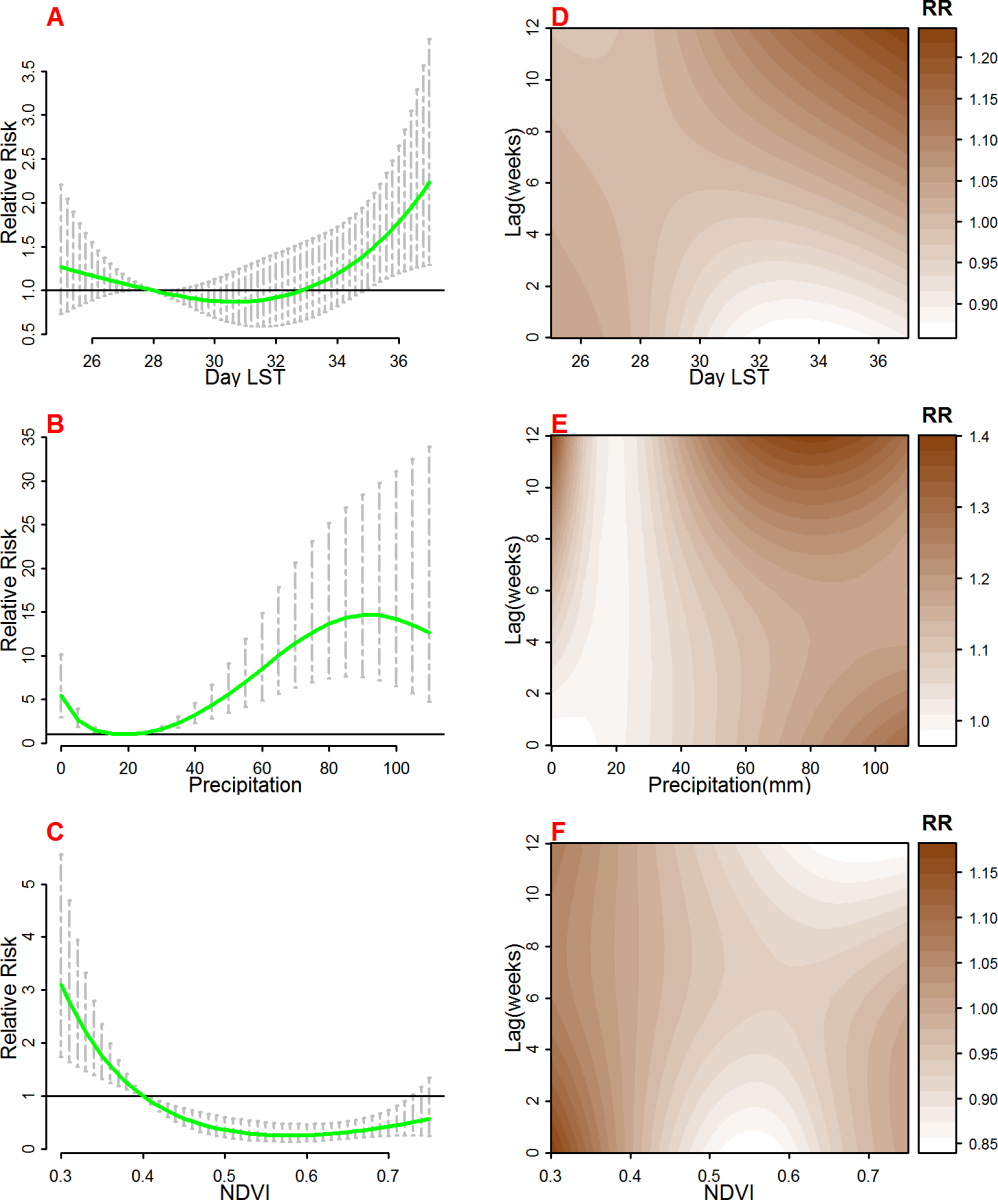
The overall risk of day Land Surface Temperature (LST °C) (A), Precipitation (mm) (B), and Normalized Difference Vegetation Index (NDVI) (C) on malaria mortality for all areas for the whole lag period. The bold lines indicate relative risks while the shaded regions display the 95% Confidence intervals. D, E and F are the lag patterns for day LST, precipitation and NDVI respectively at whole range of predictors.

The overall effects of each of the remotely sensed variables on malaria mortality and lag patterns are presented in Fig 3 and S5 Fig for monthly seasonally adjusted results. The results are combined for all three areas. For day LST (Fig 3A), in comparison with the reference temperature of 28°C, we observed increased risk at temperatures below the reference and above the reference forming a U-shaped relationship which is significant for day LST between 34°C and 36°C. When monthly seasonal effect is included, Day LST above 28°C show protective effect on malaria mortality as displayed in S5A Fig. The lag effect of LST (Fig 3D) is distributed with shorter delays at weekly mean temperatures below 28°C, while at longer delays, particularly after 9 weeks with temperature above 28°C appear more influential.

The overall effect of rainfall (Fig 3B) on malaria mortality showed increased risk with increasing rainfall above the reference point of 20 mm and peaking at about 80mm per week. Precipitation below 20mm per week also indicated increasing risks of mortality. Furthermore, there was increase in risk of mortality for weekly precipitation of above 80mm in the seasonally adjusted models shown in S5B Fig. There were delayed effects on mortality with precipitation amounts below 20 mm, while much shorter lag effect was observed with precipitation above 80mm (Fig 3E).

The overall effect of vegetation index (Fig 3C) was highest with NDVI values below 0.4 which was the reference point. The risk increased with decreasing NDVI values with highest relative risk observed at 0.3. NDVI values above 0.4 were negatively associated with malaria mortality with significant relative risks below 1. We do not observe effects of NDVI in the seasonally adjusted model displayed in S5C Fig.

The lag effect (Fig 3F) was shorter with NDVI values below 0.4 with effects from zero weeks up to 4 weeks and then increasing again after 8 weeks. The effect of NDVI above 0.4 showed consistent pattern across the weeks.

**Fig 4:**
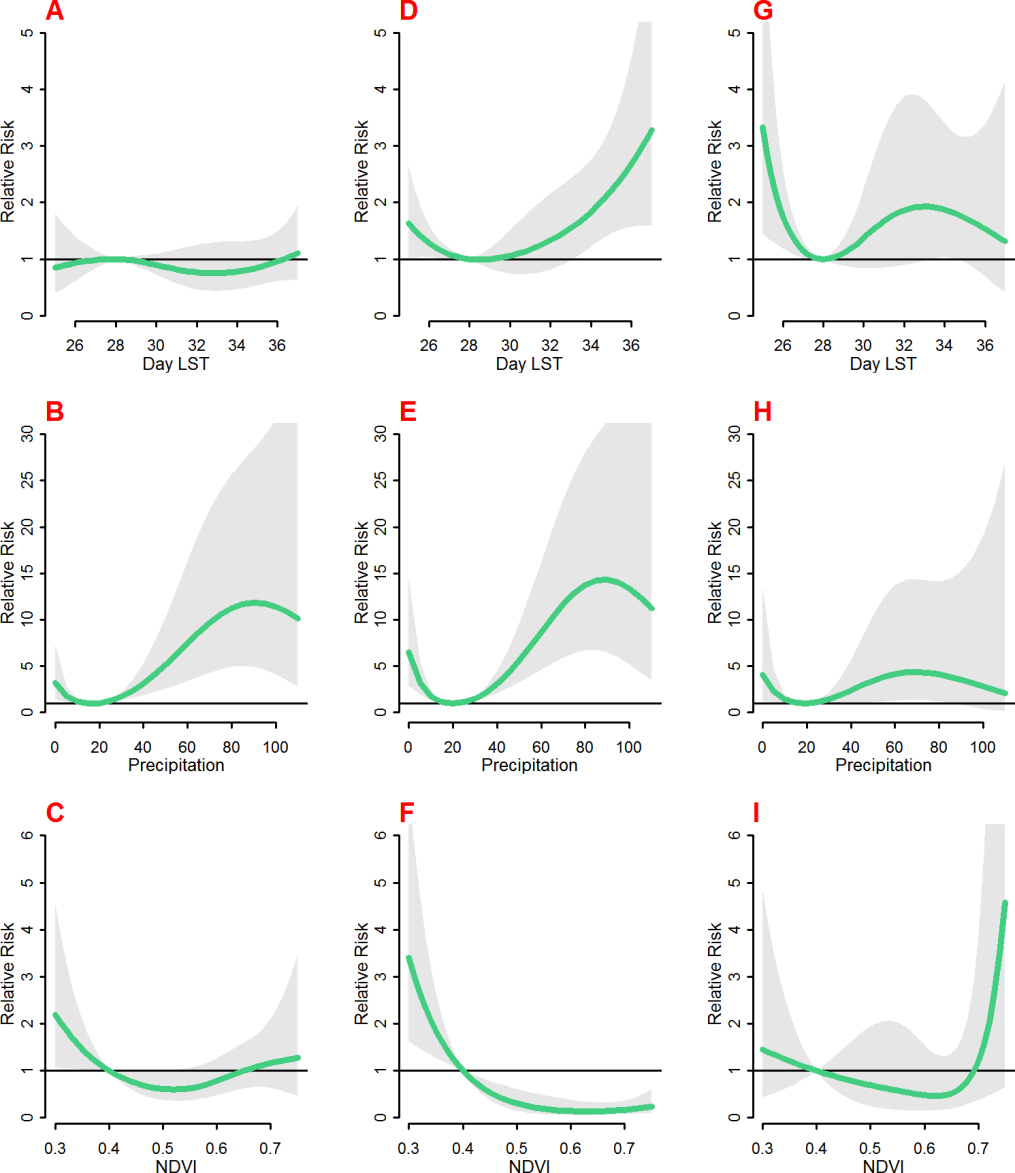
The overall risk of day LST (°C) (A, D and G), Precipitation (mm) (B, E and H), and NDVI (C, F and I) on malaria mortality in Asembo (A, B and C), Gem (D, E and F) and Karemo (G, H and I) for the whole lag period. The bold lines indicate relative risks while the shaded regions display the 95% Confidence intervals.

Fig 4 shows the overall effect of each of the remote sensing variable on malaria mortality by area while S6 Fig shows the results for models with monthly seasonal adjustments. The day LST effect varies between the three areas with no effect observed in Asembo (Fig 4A), Gem (Fig 4D) above 33°C and Karemo below 28°C (Fig 4G). For the seasonal adjusted results, there is decreased risk with Day LST above 28°C in Asembo (S6A Fig), decreased risk between 28°C and 34°C in Gem (S6D Fig) and high risk below 28°C in Karemo (S6G Fig).

Precipitation effect was very consistent between the areas with risk above 20mm with peaks at about 80mm of weekly precipitation (Fig 4B, 4E and 4H). The seasonally adjusted results show marginally significant risks in Asembo and Gem for precipitation above 70mm (S6 b and e) and risk for weekly precipitation of between 40mm and 80mm in Karemo (S6 h).

The effect of vegetation cover was very consistent in the three areas with higher risks of mortality with NDVI below 0.4 and negative association for NDVI above 0.4 (Fig 4C, 4F and 4I). There was no effect of NDVI in the seasonally adjusted models in Gem and Karemo (S6 f and i) and marginal effect of NDVI values of about 0.66 to 0.7 in Asembo (S6 c).

There was negative relationship between weekly mean LST of 29°C in the earlier weeks in Asembo and Gem, and for all areas combined as displayed in S1 Table. However, the overall effect of weekly mean day LST of 29°C is not significant in all the three areas. The overall effect of precipitation across most lags is significant with the highest overall relative risk of 1.68 (CI: 1.31–2.14) in Asembo and lowest in Gem with relative risk of 1.49 (CI: 1.28–1.74). The association and lag pattern between malaria mortality and precipitation showed the most consistent pattern between the three different study areas.

The lag structure of weekly vegetation index of 0.3 compared to 0.4 was significantly higher in week 3 and 4 in Asembo, weeks 0 to 4 in Gem with no effect in Karemo. The overall effect across all weeks was significant in all the areas with highest in Gem at relative risk of 3.4(CI: 1.64–7.05) and lowest in Asembo at 2.18(CI: 1.06–4.51).

## Discussion

We have modelled the exposure response relationship between three remote sensing variables (LST, precipitation and NDVI) and malaria mortality in three areas in Western Kenya using distributed lag non-linear modelling approach. The flexibility of the modelling framework allowed us to model both the exposure effect as well as the lag effect taking into account non-linear structure of the relationship.

The results showed non-linear relationships and delayed effects between remote sensing data and malaria mortality that were consistent between the three study areas in Western Kenya, and describe how the meteorological patterns partly drive vegetation change and precede malaria mortality in the study region. This corroborates the expected biological interactions between vectors, parasites and human hosts generating malaria incidence, morbidity, and mortality [9, 10]. The DLNM approach has been used in malaria weather epidemiology in other settings [32, 33].

Since mosquito density is important in understanding malaria transmission dynamics, temperature is one of the crucial factors influencing mosquito development. Using compartmental models, it has been shown that at 17 to 33°C, vector abundance is stable, corresponding to endemic transmission as in the study area. However, at temperatures of 20 to 26°C model equilibrium is distorted corresponding to upsurge in mosquito vector population [34].

Larval temperature has also been shown to affect adult mosquito survival with increased temperature resulting in increased mortality during larval stage. For example, an increase in temperature by 4°C from 23 to 27°C resulted in significant increase in mortality [35].

Our findings show LST to be negatively associated with malaria mortality. Negative effect of day LST on malaria mosquito density at one month lag was shown in the study area [36]. For temperatures above 28°C, we observe positive relationship between day LST and malaria mortality with lag effect after two months. In Argentina [37], LST was shown to be the most important driver of malaria vector abundance however this study did not consider the lagged effect of LST. It has been shown that in hot weather, an increase in temperature in certain ranges results in rapid development of the malaria vectors and thus expected subsequent increase in mortality [34]. The overall effect of LST on malaria mortality conforms with the optimal temperature for malaria vector development, which are between 25°C and 30°C [16]. We note, however, that LST was shown not to have any effect on sporozite rate [38]. Previous analysis showed a more delayed effect of weekly mean temperatures on malaria mortality after week 9 in the study area [11]. This is similar to our finding for the lag pattern of temperatures above 28°C, however, in this analysis we used daytime LST, which is a proxy for maximum temperature.

The overall effect of LST was different in the three areas, with increased risk for malaria mortality with LST above 33°C in Gem, while in Karemo effect was for LST below 28°C. This may be due to the fact that Gem has lower temperatures compared to the other areas at much higher altitude so an increase in temperature results in faster development of the vectors while in Karemo which is relatively hotter, temperature thresholds of 25–28°C drives vector development. The exposure response relationship between day LST and risk of malaria mortality was different when we included smooth seasonal adjustment into the model. The relationship is negative for temperatures above 28°C and no significant effect for temperatures below 28°C. This can be explained by the possible interaction between the monthly seasonal component and day LST.

The effect of rainfall was very consistent in the three areas of the HDSS with very similar lag patterns and positive association with malaria mortality across lag periods. This could be explained by the fact that the seasonal patterns of rainfall are similar in the three regions and partly by the fact that the TRMM satellite covers large area at 0.25° by 0.25° spatial resolution. This may mask the differences between the three areas due to overlapping grid points. However, our results are very similar to other studies, with increasing risk having increasing lags in hot weather countries as observed in China [32], and the sinusoidal relationship as exemplified [33] for *P*. *falciparum* malaria in China with increased risk below 20mm and above 40mm of weekly rainfall with peaks at approximately 80mm. The lag structure found in the present study was also similar for areas described as hot in Ethiopia with lag effect after six weeks [39] and 1–47 days in Kenyan coast [40]. We observed an almost linear increase in risk of malaria mortality with the addition of seasonal component in the precipitation model parameters. Its significant for weeks with over 80mm of rainfall. The seasonal component may have masked the overall risk pattern.

Vegetation characteristics provide different opportunities for vectors to thrive e.g. crop type determines breeding and resting place for mosquitoes, greening of vegetation determines timing of habitat creation and deforestation results in sunlit pools suitable for breeding [41].

The overall effect of vegetation index NDVI in our study corroborates similar findings in Coastal region of Kenya that an NDVI threshold of 0.3–0.4 [20, 42] is a necessary condition to drive increase in malaria incidence. In the previous study at the Kenyan Coast, a one month lag of NDVI was shown to be highly correlated (R^2^ = 0.71) with monthly proportion of annual malaria cases which is consistent with our finding of an effect with 0 to 4 weeks for NDVI below 0.4. Similar results of these NDVI values corresponding to high malaria transmission have also been observed in Bangladesh [43].

In the present study, NDVI values above 0.4 were negatively associated with mortality. Because NDVI is associated with lag patterns of rainfall, higher NDVI corresponds to higher previous precipitation weeks which may result in flashing of the mosquito vector larvae leading to decreased incidence. The exposure relationship was similar in the three areas with lag effect strongest at 0 to 4 weeks in Gem and Karemo which are consistent with a study in Ethiopian highlands [44] and lag effect in week 3 in Karemo, however the overall effect was significant for all the three areas. NDVI was highly correlated with rainfall with lags of 1 to 4 weeks in both Asembo and Karemo while in Gem after 5–8 weeks. This is consistent with expectation of increases in green vegetation cover after weeks of rainfall. Similar findings have been shown in Iran [45].

The present study had certain limitations. The study uses Inter-VA Verbal Autopsy methodology to extract malaria-specific cause of deaths from Verbal Autopsy questionnaire. This is a statistical method that may under-estimate or over-estimate malaria deaths in the region. In low income countries such as Kenya, this is the most efficient way of ascertaining cause of death where vital registration is incomplete. The Inter-VA method for ascertaining cause of death has been shown to have high concordance with physician coding [25] and malaria deaths derived using the methodology correlates well with Malaria Atlas projections [46].

Our analysis also does not take into account other interventions that have been implemented in the HDSS area over the years and human activities and prevention strategies that would have explained differences in mortality besides the environmental variables. There are intrinsic factors, crucial in determining vertebrate host immunity and important to consider together with the environmental factors, but were beyond the scope of this study. The inclusion of the trend variable alone may not capture all shorter-term influence of such unmeasured confounders. Another limitation is the assumption of linearity for the results, and use of same centering values and lag structure in the three areas that have different environmental patterns.

## Conclusions

This study described association of lag patterns of weekly remotely sensed environmental and meteorological conditions on malaria mortality in three malaria endemic regions in Western Kenya. The estimates in the present study provide information on the degree to which these conditions drive the mortality patterns in an area of stable malaria transmission, and indicate suitable lead times for the development of malaria early warning systems in malaria endemic regions. The analysis shows how use of remote sensing could help in the forecast and control of malaria, as has been articulated in this study [47]. Our findings corroborate expected biological mechanism of the development of the vector as well as the parasite in the vector related to environmental factors, but also indicate some geographical dissimilarities in the associations. The results provide further evidence in the body of literature on lagged association of weather factors and malaria. Our study also reinforces the applicability of the use of remote-sensing data which is available at appropriate spatial and temporal resolution for risk mapping of malaria in areas where weather data is not reliable and readily available.

## Supporting Information

S1 Table Malaria mortality Relative Risks and 95% confidence intervals in brackets by area for 1°C increase in day LST, 10 mm increase in Precipitation (mm) and 0.1 decrease in NDVI below the reference point for each lag weeks. (DOCX)

S1 Fig Lag pattern and association of precipitation and NDVI. (TIFF)

S2 Fig Cross-correlations coefficients between LST and NDVI, Precipitation and NDVI and precipitation and LST. (TIFF)

S3 Fig Contour plots show showing Relative Risks of malaria mortality at different weekly lags and range of predictor variables for LST(°C) (A, D and G), precipitation(mm) (B, E and H) and NDVI (C, F and I) in Asembo (A, B and C), Gem (D, E and F) and Karemo (G, H and I)…
(TIFF)

S4 Fig Monthly seasonal pattern of mortality in Asembo,Gem and karemo 2003–2013. (TIFF)

S5 Fig The overall Risk of day Land Surface Temperature (LST °C) (A), Precipitation (mm) (B), and Normalized Difference Vegetation Index (NDVI) (C) on malaria mortality for all areas for the whole lag period including smooth of month of year to adjust for seasonality. The bold lines indicate Relative risks while the shaded regions display the 95% Confidence intervals. D, E and F are the lag patterns for day LST, precipitation and NDVI respectively at whole range of predictors.
(TIFF)

S6 Fig The overall Risk of day LST (°C) (A, D and G), Precipitation (mm) (B, E and H), and NDVI (C, F and I) on malaria mortality in Asembo (A, B and C), Gem (D, E and F) and Karemo (G, H and I) for the whole lag period including smooth of month of year to adjust for seasonality. The bold lines indicate Relative risks while the shaded regions display the 95% Confidence intervals.
(TIFF)

## References

[1] WHO. World Malaria Report 2013.

[2] Desai M, Buff AM, Khagayi S, Byass P, Amek N, van Eijk A, et al Age-Specific Malaria Mortality Rates in the KEMRI/CDC Health and Demographic Surveillance System in Western Kenya, 2003–2010. PloS one. 2014;9(9):e106197 Epub 2014/09/03. 10.1371/journal.pone.0106197 ; PubMed Central PMCID: PMCPmc4152016. 25180495 PMC4152016

[3] Amek NO, Odhiambo FO, Khagayi S, Moige H, Orwa G, Hamel MJ, et al Childhood cause-specific mortality in rural Western Kenya: application of the InterVA-4 model. Global health action. 2014;7:25581 Epub 2014/11/08. 10.3402/gha.v7.25581 ; PubMed Central PMCID: PMCPmc4221497. 25377340 PMC4221497

[4] Ehlkes L, Krefis AC, Kreuels B, Krumkamp R, Adjei O, Ayim-Akonor M, et al Geographically weighted regression of land cover determinants of Plasmodium falciparum transmission in the Ashanti Region of Ghana. International journal of health geographics. 2014;13(1):35 Epub 2014/10/02. 10.1186/1476-072x-13-35 . 25270342 PMC4192530

[5] Krefis AC, Schwarz NG, Nkrumah B, Acquah S, Loag W, Oldeland J, et al Spatial analysis of land cover determinants of malaria incidence in the Ashanti Region, Ghana. PloS one. 2011;6(3):e17905 Epub 2011/03/31. 10.1371/journal.pone.0017905 ; PubMed Central PMCID: PMCPmc3063166. 21448277 PMC3063166

[6] McCann RS, Messina JP, MacFarlane DW, Bayoh MN, Vulule JM, Gimnig JE, et al Modeling larval malaria vector habitat locations using landscape features and cumulative precipitation measures. International journal of health geographics. 2014;13:17 Epub 2014/06/07. 10.1186/1476-072x-13-17 ; PubMed Central PMCID: PMCPmc4070353. 24903736 PMC4070353

[7] Cohen JM, Ernst KC, Lindblade KA, Vulule JM, John CC, Wilson ML. Local topographic wetness indices predict household malaria risk better than land-use and land-cover in the western Kenya highlands. Malaria journal. 2010;9:328 Epub 2010/11/18. 10.1186/1475-2875-9-328 ; PubMed Central PMCID: PMCPmc2993734. 21080943 PMC2993734

[8] Mutuku FM, Bayoh MN, Hightower AW, Vulule JM, Gimnig JE, Mueke JM, et al A supervised land cover classification of a western Kenya lowland endemic for human malaria: associations of land cover with larval Anopheles habitats. International journal of health geographics. 2009;8:19 Epub 2009/04/18. 10.1186/1476-072x-8-19 ; PubMed Central PMCID: PMCPmc2676261. 19371425 PMC2676261

[9] Craig MH, Snow RW, le Sueur D. A climate-based distribution model of malaria transmission in sub-Saharan Africa. Parasitology today (Personal ed). 1999;15(3):105–11. Epub 1999/05/14. . 10322323 10.1016/s0169-4758(99)01396-4

[10] Snow RW, Gouws E, Omumbo J, Rapuoda B, Craig MH, Tanser FC, et al Models to predict the intensity of Plasmodium falciparum transmission: applications to the burden of disease in Kenya. Transactions of the Royal Society of Tropical Medicine and Hygiene. 1998;92(6):601–6. Epub 1999/05/18. . 10326100 10.1016/s0035-9203(98)90781-7

[11] Sewe M, Rocklov J, Williamson J, Hamel M, Nyaguara A, Odhiambo F, et al The Association of Weather Variability and Under Five Malaria Mortality in KEMRI/CDC HDSS in Western Kenya 2003 to 2008: A Time Series Analysis. International journal of environmental research and public health. 2015;12(2):1983–97. 10.3390/ijerph120201983 . 25674784 PMC4344705

[12] Connor SJ, Ceccato P, Dinku T, Omumbo J, Grover-Kopec EK, Thomson MC. Using climate information for improved health in Africa: relevance, constraints and opportunities. Geospatial health. 2006;1(1):17–31. Epub 2008/08/08. . 18686230 10.4081/gh.2006.278

[13] Brown ME, Grace K, Shively G, Johnson KB, Carroll M. Using satellite remote sensing and household survey data to assess human health and nutrition response to environmental change. Population and environment. 2014;36:48–72. Epub 2014/08/19. 10.1007/s11111-013-0201-0 ; PubMed Central PMCID: PMCPmc4131131. 25132700 PMC4131131

[14] Ceccato P, Connor SJ, Jeanne I, Thomson MC. Application of Geographical Information Systems and Remote Sensing technologies for assessing and monitoring malaria risk. Parassitologia. 2005;47(1):81–96. Epub 2005/07/28. . 16044677

[15] Kalluri S, Gilruth P, Rogers D, Szczur M. Surveillance of arthropod vector-borne infectious diseases using remote sensing techniques: a review. PLoS pathogens. 2007;3(10):1361–71. Epub 2007/10/31. 10.1371/journal.ppat.0030116 ; PubMed Central PMCID: PMCPmc2042005. 17967056 PMC2042005

[16] Thomson MC, Connor SJ, Milligan P, Flasse SP. Mapping malaria risk in Africa: What can satellite data contribute? Parasitology today (Personal ed). 1997;13(8):313–8. Epub 1997/08/01. . 15275058 10.1016/s0169-4758(97)01097-1

[17] Omumbo JA, Hay SI, Goetz SJ, Snow RW, Rogers DJ. Updating Historical Maps of Malaria Transmission Intensity in East Africa Using Remote Sensing. Photogrammetric engineering and remote sensing. 2002;68(2):161–6. Epub 2002/02/01. ; PubMed Central PMCID: PMCPmc3694357. 23814324 PMC3694357

[18] Omumbo JA, Hay SI, Snow RW, Tatem AJ, Rogers DJ. Modelling malaria risk in East Africa at high-spatial resolution. Tropical medicine & international health: TM & IH. 2005;10(6):557–66. Epub 2005/06/09. 10.1111/j.1365-3156.2005.01424.x ; PubMed Central PMCID: PMCPmc3191364. 15941419 PMC3191364

[19] Clennon JA, Kamanga A, Musapa M, Shiff C, Glass GE. Identifying malaria vector breeding habitats with remote sensing data and terrain-based landscape indices in Zambia. International journal of health geographics. 2010;9:58 Epub 2010/11/06. 10.1186/1476-072x-9-58 ; PubMed Central PMCID: PMCPmc2993656. 21050496 PMC2993656

[20] Hay SI, Snow RW, Rogers DJ. From predicting mosquito habitat to malaria seasons using remotely sensed data: practice, problems and perspectives. Parasitology today (Personal ed). 1998;14(8):306–13. Epub 2006/10/17. . 17040796 10.1016/s0169-4758(98)01285-x

[21] Kelly-Hope LA, Hemingway J, McKenzie FE. Environmental factors associated with the malaria vectors Anopheles gambiae and Anopheles funestus in Kenya. Malaria journal. 2009;8:268 Epub 2009/11/28. 10.1186/1475-2875-8-268 ; PubMed Central PMCID: PMCPmc2793260. 19941637 PMC2793260

[22] Odhiambo FO, Laserson KF, Sewe M, Hamel MJ, Feikin DR, Adazu K, et al Profile: the KEMRI/CDC Health and Demographic Surveillance System—Western Kenya. International journal of epidemiology. 2012;41(4):977–87. Epub 2012/08/31. 10.1093/ije/dys108 . 22933646 PMC12083774

[23] Adazu K, Lindblade KA, Rosen DH, Odhiambo F, Ofware P, Kwach J, et al Health and demographic surveillance in rural western Kenya: a platform for evaluating interventions to reduce morbidity and mortality from infectious diseases. The American journal of tropical medicine and hygiene. 2005;73(6):1151–8. Epub 2005/12/16. . 16354829

[24] Byass P, Chandramohan D, Clark SJ, D'Ambruoso L, Fottrell E, Graham WJ, et al Strengthening standardised interpretation of verbal autopsy data: the new InterVA-4 tool. Global health action. 2012;5:1–8. Epub 2012/09/05. 10.3402/gha.v5i0.19281 ; PubMed Central PMCID: PMCPmc3433652. 22944365 PMC3433652

[25] Byass P, Herbst K, Fottrell E, Ali MM, Odhiambo F, Amek N, et al Comparing verbal autopsy cause of death findings as determined by physician coding and probabilistic modelling: a public health analysis of 54 000 deaths in Africa and Asia. J Glob Health. 2015;5(1):010402 Epub 2015/03/04. 10.7189/jogh.05.010402 25734004 PMC4337147

[26] USGS. Available: https://lpdaac.usgs.gov/.

[27] Rowlingson RBaTKaB. rgdal: Bindings for the Geospatial Data Abstraction Library. 2014.

[28] Hallman J. tis: Time Indexes and Time Indexed Series 2013. Available: http://CRAN.R-project.org/package=tis.

[29] R Core Team R: A language and environment for statistical computing. R Foundation for Statistical Computing, Vienna, Austria 2014 Available: http://www.R-project.org/.

[30] Gasparrini A. Distributed Lag Linear and Non-Linear Models in R: The Package dlnm. Journal of statistical software. 2011;43(8):1–20. Epub 2011/10/18. ; PubMed Central PMCID: PMCPmc3191524. 22003319 PMC3191524

[31] Gasparrini A. Modeling exposure-lag-response associations with distributed lag non-linear models. Statistics in medicine. 2014;33(5):881–99. Epub 2013/09/13. 10.1002/sim.5963 ; PubMed Central PMCID: PMCPmc4098103. 24027094 PMC4098103

[32] Zhao X, Chen F, Feng Z, Li X, Zhou XH. The temporal lagged association between meteorological factors and malaria in 30 counties in south-west China: a multilevel distributed lag non-linear analysis. Malaria journal. 2014;13:57 Epub 2014/02/18. 10.1186/1475-2875-13-57 24528891 PMC3932312

[33] Bi Y, Yu W, Hu W, Lin H, Guo Y, Zhou XN, et al Impact of climate variability on Plasmodium vivax and Plasmodium falciparum malaria in Yunnan Province, China. Parasites & vectors. 2013;6:357 Epub 2013/12/18. 10.1186/1756-3305-6-357 24341555 PMC3898806

[34] Beck-Johnson LM, Nelson WA, Paaijmans KP, Read AF, Thomas MB, Bjornstad ON. The effect of temperature on Anopheles mosquito population dynamics and the potential for malaria transmission. PloS one. 2013;8(11):e79276 Epub 2013/11/19. 10.1371/journal.pone.0079276 ; PubMed Central PMCID: PMCPmc3828393. 24244467 PMC3828393

[35] Christiansen-Jucht C, Parham PE, Saddler A, Koella JC, Basanez MG. Temperature during larval development and adult maintenance influences the survival of Anopheles gambiae s.s. Parasites & vectors. 2014;7:489 Epub 2014/11/05. 10.1186/s13071-014-0489-3 ; PubMed Central PMCID: PMCPmc4236470. 25367091 PMC4236470

[36] Amek N, Bayoh N, Hamel M, Lindblade KA, Gimnig JE, Odhiambo F, et al Spatial and temporal dynamics of malaria transmission in rural Western Kenya. Parasites & vectors. 2012;5:86 Epub 2012/05/01. 10.1186/1756-3305-5-86 ; PubMed Central PMCID: PMCPmc3464956. 22541138 PMC3464956

[37] Dantur Juri MJ, Estallo E, Almiron W, Santana M, Sartor P, Lamfri M, et al Satellite-derived NDVI, LST, and climatic factors driving the distribution and abundance of Anopheles mosquitoes in a former malarious area in northwest Argentina. Journal of vector ecology: journal of the Society for Vector Ecology. 2015;40(1):36–45. Epub 2015/06/06. 10.1111/jvec.12130 . 26047182

[38] Amek N, Bayoh N, Hamel M, Lindblade KA, Gimnig J, Laserson KF, et al Spatio-temporal modeling of sparse geostatistical malaria sporozoite rate data using a zero inflated binomial model. Spatial and spatio-temporal epidemiology. 2011;2(4):283–90. Epub 2012/07/04. 10.1016/j.sste.2011.08.001 . 22748226

[39] Teklehaimanot HD, Lipsitch M, Teklehaimanot A, Schwartz J. Weather-based prediction of Plasmodium falciparum malaria in epidemic-prone regions of Ethiopia I. Patterns of lagged weather effects reflect biological mechanisms. Malaria journal. 2004;3:41 Epub 2004/11/16. 10.1186/1475-2875-3-41 15541174 PMC535540

[40] Walker M, Winskill P, Basanez MG, Mwangangi JM, Mbogo C, Beier JC, et al Temporal and micro-spatial heterogeneity in the distribution of Anopheles vectors of malaria along the Kenyan coast. Parasites & vectors. 2013;6:311 Epub 2013/12/18. 10.1186/1756-3305-6-311 ; PubMed Central PMCID: PMCPmc3843567. 24330615 PMC3843567

[41] Beck LR, Lobitz BM, Wood BL. Remote sensing and human health: new sensors and new opportunities. Emerging infectious diseases. 2000;6(3):217–27. Epub 2000/05/29. 10.3201/eid0603.000301 ; PubMed Central PMCID: PMCPmc2640871. 10827111 PMC2640871

[42] Hay SI, Snow RW, Rogers DJ. Predicting malaria seasons in Kenya using multitemporal meteorological satellite sensor data. Transactions of the Royal Society of Tropical Medicine and Hygiene. 1998;92(1):12–20. Epub 1998/08/06. . 9692138 10.1016/s0035-9203(98)90936-1

[43] Haque U, Hashizume M, Glass GE, Dewan AM, Overgaard HJ, Yamamoto T. The role of climate variability in the spread of malaria in Bangladeshi highlands. PloS one. 2010;5(12):e14341 10.1371/journal.pone.0014341 21179555 PMC3002939

[44] Midekisa A, Senay G, Henebry GM, Semuniguse P, Wimberly MC. Remote sensing-based time series models for malaria early warning in the highlands of Ethiopia. Malaria journal. 2012;11:165 Epub 2012/05/16. 10.1186/1475-2875-11-165 ; PubMed Central PMCID: PMCPmc3493314. 22583705 PMC3493314

[45] Hashemi SA. Investigation of Relationship Between Rainfall and Vegetation Index by Using NOAA/AVHRR Satellite Images. World Applied Sciences Journal. 2011;14(11): 1678–82.

[46] Streatfield PK, Khan WA, Bhuiya A, Hanifi SM, Alam N, Diboulo E, et al Malaria mortality in Africa and Asia: evidence from INDEPTH health and demographic surveillance system sites. Global health action. 2014;7:25369 Epub 2014/11/08. 10.3402/gha.v7.25369 25377329 PMC4220130

[47] Rogers DJ, Randolph SE, Snow RW, Hay SI. Satellite imagery in the study and forecast of malaria. Nature. 2002;415(6872):710–5. Epub 2002/02/08. 10.1038/415710a ; PubMed Central PMCID: PMCPmc3160466. 11832960 PMC3160466

