# Supplementary material for: Remotely Sensed Environmental Conditions and Malaria Mortality in Three Malaria Endemic Regions in Western Kenya
Source: PLoS One. 2016 Apr 26;11(4):e0154204. doi: 10.1371/journal.pone.0154204 (PMC4845989; doi:10.1371/journal.pone.0154204)
Supplement: S1 Table — (DOCX) [file pone.0154204.s001.docx]

S1 Table. Malaria mortality Relative Risks and 95% confidence intervals in brackets by area for 1^o^ C increase in day LST, 10 mm increase in Precipitation (mm) and 0.1 decrease in NDVI below the reference point for each lag weeks.

| Lag | Asembo | Gem | Karemo | All Areas |
| --- | --- | --- | --- | --- |
| Weeks | *Land Surface Temperature* | | | |
| 0 | 0.98(0.96,1) | 0.95(0.92,0.98)* | 0.99(0.94,1.04) | 0.96(0.94,0.98)* |
| 1 | 0.98(0.97,1) | 0.96(0.94,0.99)* | 0.99(0.96,1.03) | 0.97(0.95,0.98)* |
| 2 | 0.98(0.97,0.99)* | 0.97(0.95,0.99)* | 0.99(0.97,1.02) | 0.97(0.96,0.99)* |
| 3 | 0.99(0.98,1) | 0.98(0.97,1) | 1(0.97,1.02) | 0.98(0.97,0.99)* |
| 4 | 0.99(0.98,1) | 0.99(0.98,1.01) | 1(0.97,1.02) | 0.99(0.97,1) |
| 5 | 0.99(0.98,1) | 1(0.98,1.02) | 1(0.97,1.03) | 0.99(0.98,1) |
| 6 | 1(0.99,1.01) | 1.01(0.99,1.03) | 1(0.97,1.03) | 1(0.98,1.01) |
| 7 | 1(0.99,1.01) | 1.02(1,1.04) | 1.01(0.98,1.04) | 1(0.99,1.01) |
| 8 | 1(0.99,1.01) | 1.02(1,1.04) | 1.01(0.99,1.04) | 1.01(0.99,1.02) |
| 9 | 1.01(1,1.02) | 1.02(1.01,1.04)* | 1.02(1,1.04) | 1.01(1,1.02) |
| 10 | 1.01(1,1.02) | 1.02(1.01,1.04)* | 1.02(1,1.05) | 1.01(1,1.03) |
| 11 | 1.01(1,1.03) | 1.02(1,1.05) | 1.03(1,1.07) | 1.02(1,1.03) |
| 12 | 1.02(1,1.04) | 1.02(0.99,1.06) | 1.04(0.99,1.09) | 1.02(1,1.05) |
| All lags | 0.97(0.88,1.06) | 1(0.84,1.19) | 1.11(0.89,1.38) | 0.93(0.84,1.02) |
|  | *Precipitation* | | | |
| 0 | 1.02(0.98,1.06) | 1.04(1.01,1.07)* | 1.02(0.95,1.09) | 1.03(1,1.06) |
| 1 | 1.02(0.99,1.05) | 1.04(1.02,1.06)* | 1.01(0.96,1.06) | 1.03(1.01,1.05)* |
| 2 | 1.02(1,1.05) | 1.04(1.02,1.05)* | 1(0.96,1.04) | 1.03(1.01,1.04)* |
| 3 | 1.03(1,1.05) | 1.03(1.02,1.05)* | 1(0.96,1.03) | 1.03(1.01,1.04)* |
| 4 | 1.03(1,1.05) | 1.03(1.01,1.05)* | 1(0.96,1.03) | 1.03(1.01,1.04)* |
| 5 | 1.03(1.01,1.06)* | 1.03(1.01,1.05)* | 1(0.96,1.04) | 1.03(1.01,1.05)* |
| 6 | 1.03(1.01,1.06)* | 1.03(1.01,1.05)* | 1(0.96,1.05) | 1.03(1.01,1.05)* |
| 7 | 1.04(1.01,1.07)* | 1.03(1.01,1.05)* | 1.01(0.97,1.05) | 1.03(1.02,1.05)* |
| 8 | 1.05(1.02,1.07)* | 1.03(1.01,1.04)* | 1.03(0.99,1.07) | 1.04(1.02,1.05)* |
| 9 | 1.05(1.03,1.08)* | 1.03(1.01,1.04)* | 1.04(1.01,1.08)* | 1.04(1.03,1.06)* |
| 10 | 1.06(1.03,1.09)* | 1.03(1.01,1.05)* | 1.07(1.02,1.11)* | 1.05(1.03,1.07)* |
| 11 | 1.07(1.03,1.1)* | 1.03(1.01,1.05)* | 1.09(1.03,1.15)* | 1.06(1.03,1.08)* |
| 12 | 1.08(1.03,1.12)* | 1.03(1,1.06) | 1.11(1.03,1.2)* | 1.06(1.03,1.1)* |
| All lags | 1.68(1.31,2.14)* | 1.49(1.28,1.74)* | 1.43(0.96,2.13) | 1.61(1.37,1.89)* |
|  | *Normalized Difference Vegetation Index* | | | |
| 0 | 1.08(0.9,1.3) | 1.2(1.03,1.4)* | 1.07(0.82,1.39) | 1.18(1.03,1.35)* |
| 1 | 1.09(0.95,1.25) | 1.16(1.03,1.31)* | 1.06(0.87,1.28) | 1.16(1.05,1.28)* |
| 2 | 1.1(1,1.2) | 1.13(1.03,1.23)* | 1.05(0.91,1.21) | 1.13(1.05,1.22)* |
| 3 | 1.1(1.03,1.19)* | 1.1(1.02,1.18)* | 1.04(0.93,1.17) | 1.11(1.05,1.18)* |
| 4 | 1.1(1.02,1.19)* | 1.07(1,1.16) | 1.03(0.92,1.17) | 1.09(1.03,1.16)* |
| 5 | 1.1(1.01,1.2)* | 1.06(0.97,1.15) | 1.03(0.9,1.18) | 1.07(1,1.15) |
| 6 | 1.09(1,1.2) | 1.05(0.96,1.14) | 1.02(0.89,1.18) | 1.06(0.99,1.14) |
| 7 | 1.08(0.99,1.18) | 1.05(0.97,1.14) | 1.02(0.89,1.17) | 1.06(0.99,1.13) |
| 8 | 1.06(0.99,1.14) | 1.06(0.98,1.14) | 1.01(0.9,1.15) | 1.06(1,1.12) |
| 9 | 1.04(0.97,1.11) | 1.07(1,1.15) | 1.01(0.9,1.14) | 1.06(1,1.12) |
| 10 | 1.01(0.93,1.11) | 1.09(1.01,1.18)* | 1.01(0.88,1.16) | 1.06(1,1.14) |
| 11 | 0.99(0.87,1.12) | 1.12(1.01,1.24)* | 1.01(0.84,1.22) | 1.07(0.98,1.17) |
| 12 | 0.96(0.81,1.14) | 1.15(1,1.31) | 1.01(0.79,1.29) | 1.08(0.95,1.22) |
| All lags | 2.18(1.06,4.51)* | 3.4(1.64,7.05)* | 1.45(0.44,4.83) | 3.1(1.73,5.56)* |

*P<0.05
