# Supplementary figures and images for: Remotely Sensed Environmental Conditions and Malaria Mortality in Three Malaria Endemic Regions in Western Kenya
Source: PLoS One. 2016 Apr 26;11(4):e0154204. doi: 10.1371/journal.pone.0154204 (PMC4845989; doi:10.1371/journal.pone.0154204)

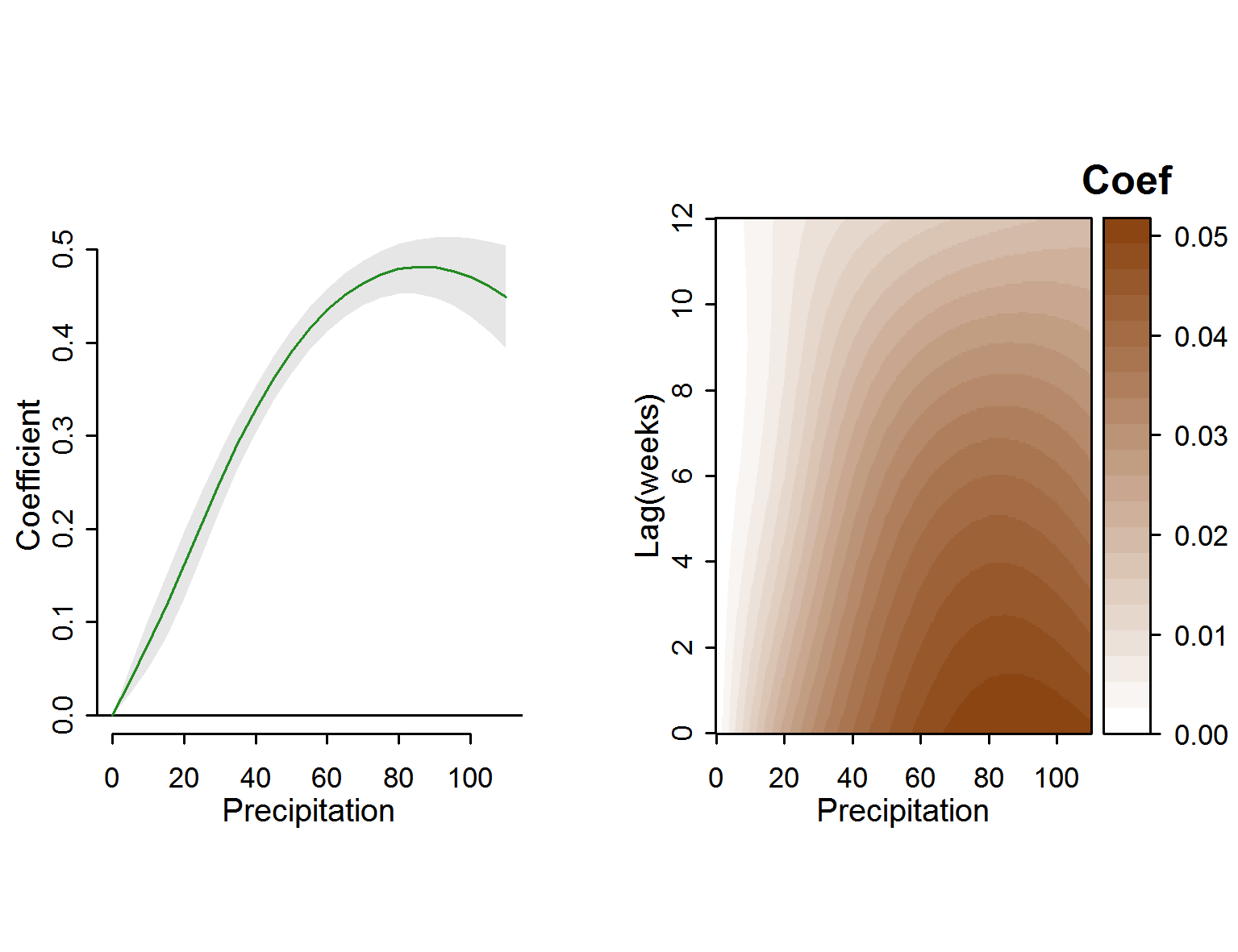

Supplement: S1 Fig — (TIFF) [file pone.0154204.s002.tiff]

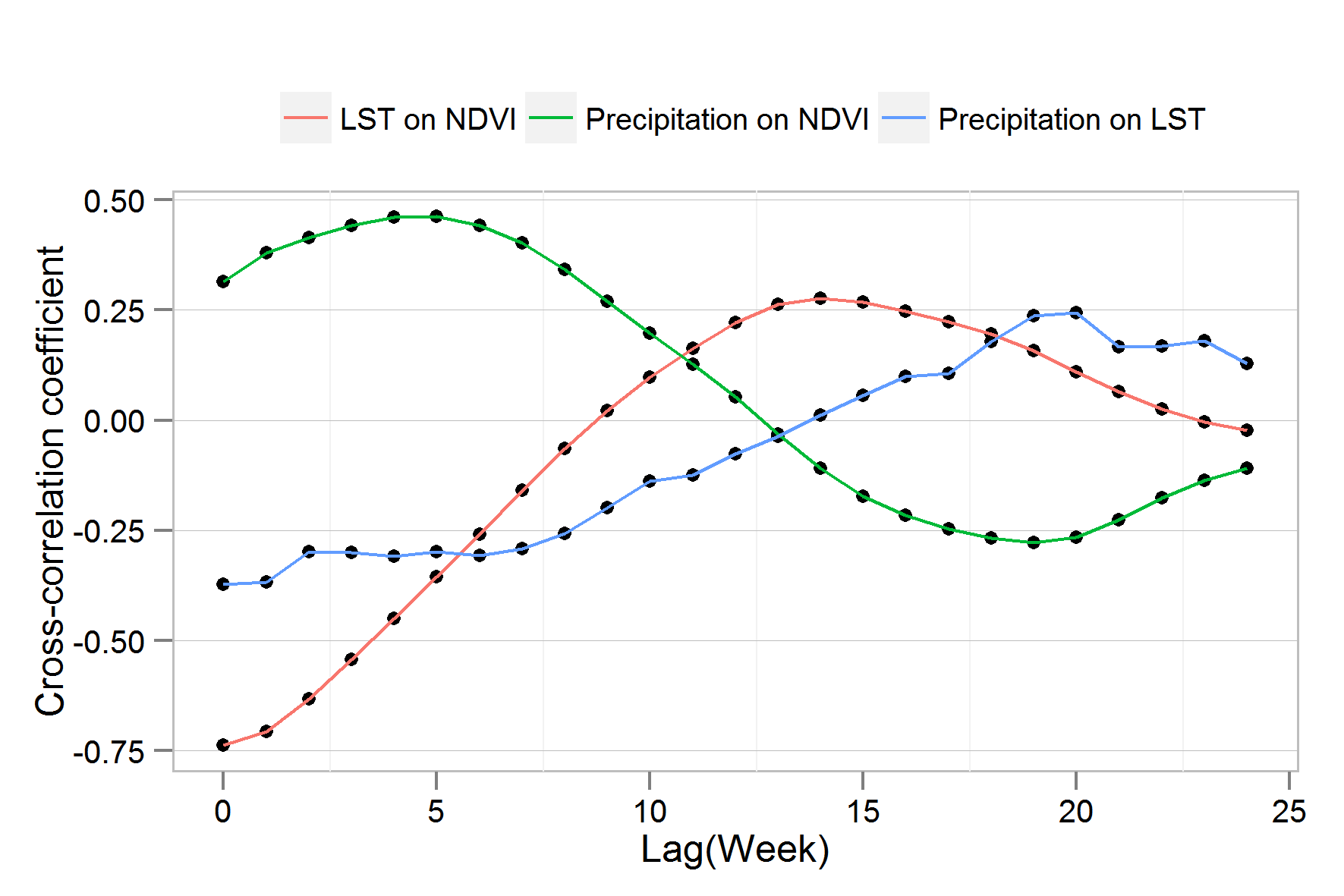

Supplement: S2 Fig — (TIFF) [file pone.0154204.s003.tiff]

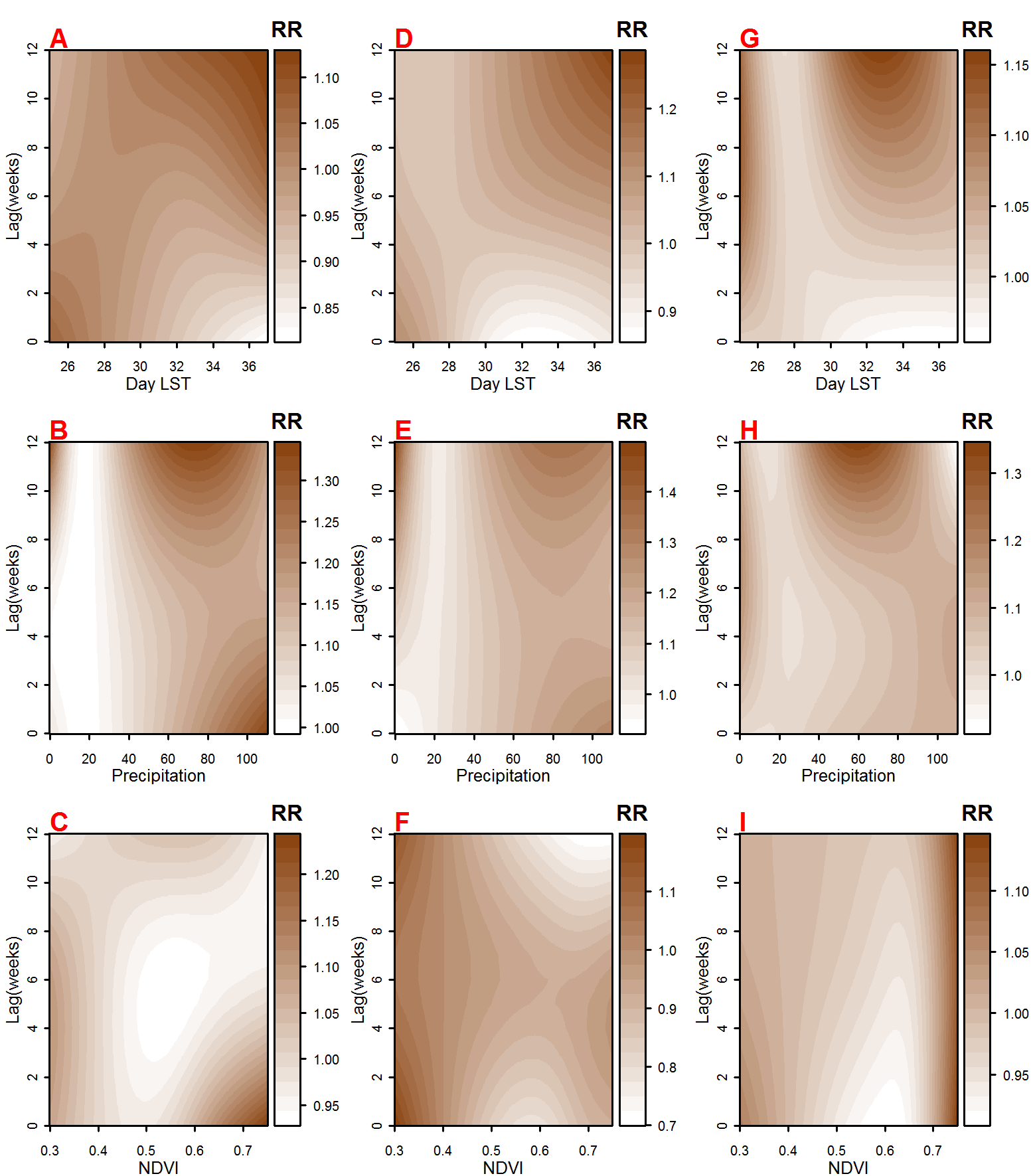

Supplement: S3 Fig — Contour plots show showing Relative Risks of malaria mortality at different weekly lags and range of predictor variables for LST(°C) (A, D and G), precipitation(mm) (B, E and H) and NDVI (C, F and I) in Asembo (A, B and C), Gem (D, E and F) and Karemo (G, H and I)… (TIFF) [file pone.0154204.s004.tiff]

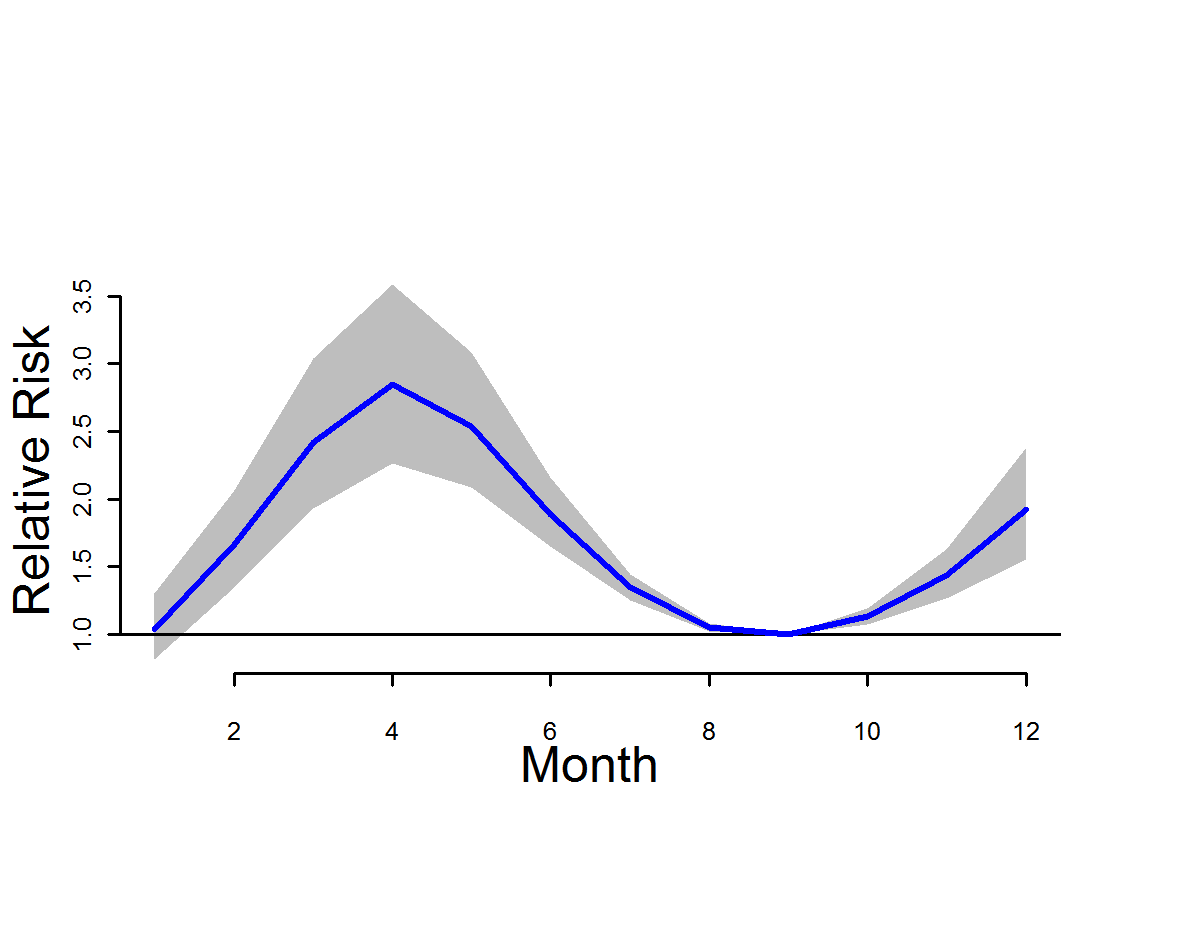

Supplement: S4 Fig — (TIFF) [file pone.0154204.s005.tiff]

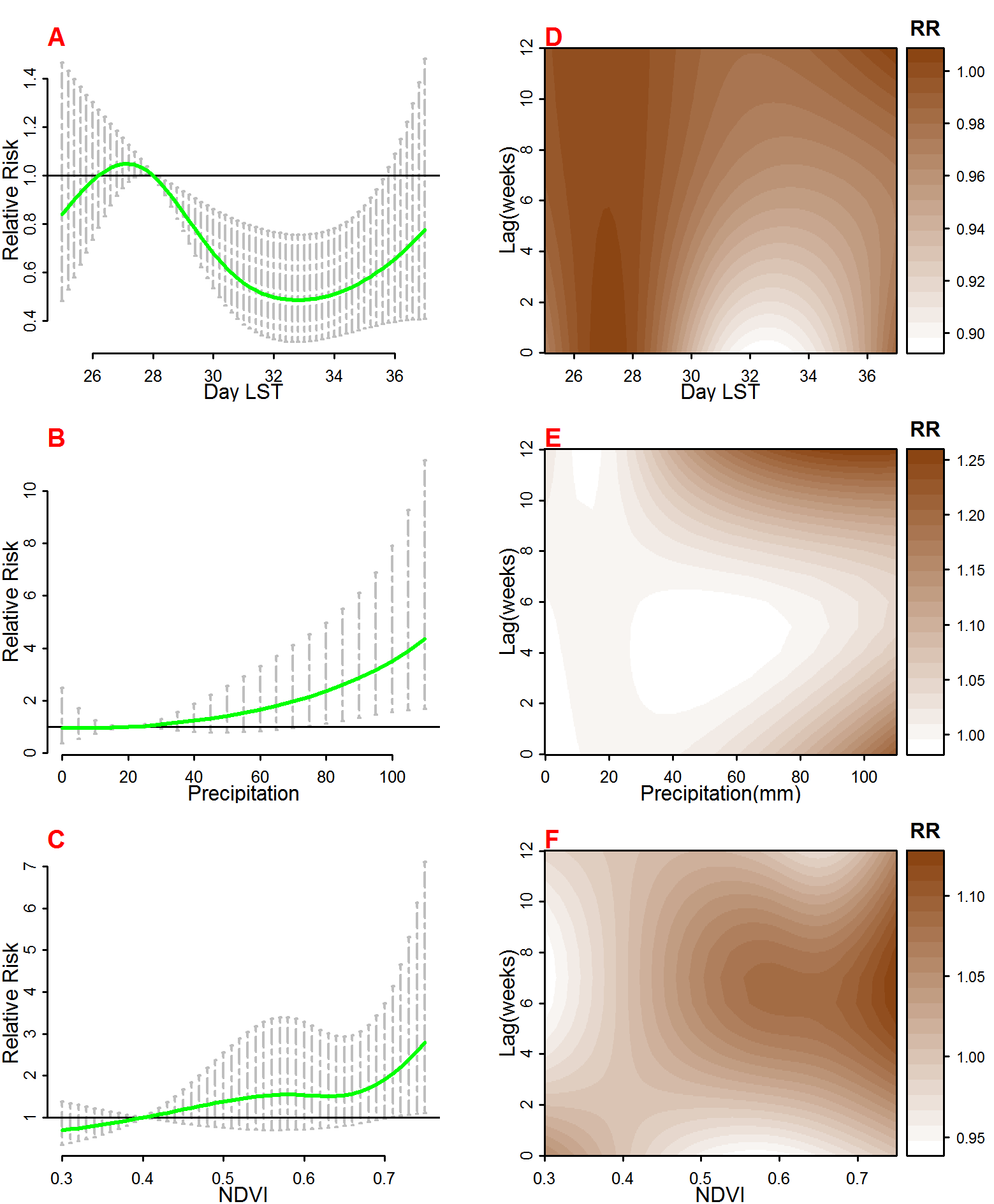

Supplement: S5 Fig — The overall Risk of day Land Surface Temperature (LST °C) (A), Precipitation (mm) (B), and Normalized Difference Vegetation Index (NDVI) (C) on malaria mortality for all areas for the whole lag period including smooth of month of year to adjust for seasonality. The bold lines indicate Relative risks while the shaded regions display the 95% Confidence intervals. D, E and F are the lag patterns for day LST, precipitation and NDVI respectively at whole range of predictors. (TIFF) [file pone.0154204.s006.tiff]

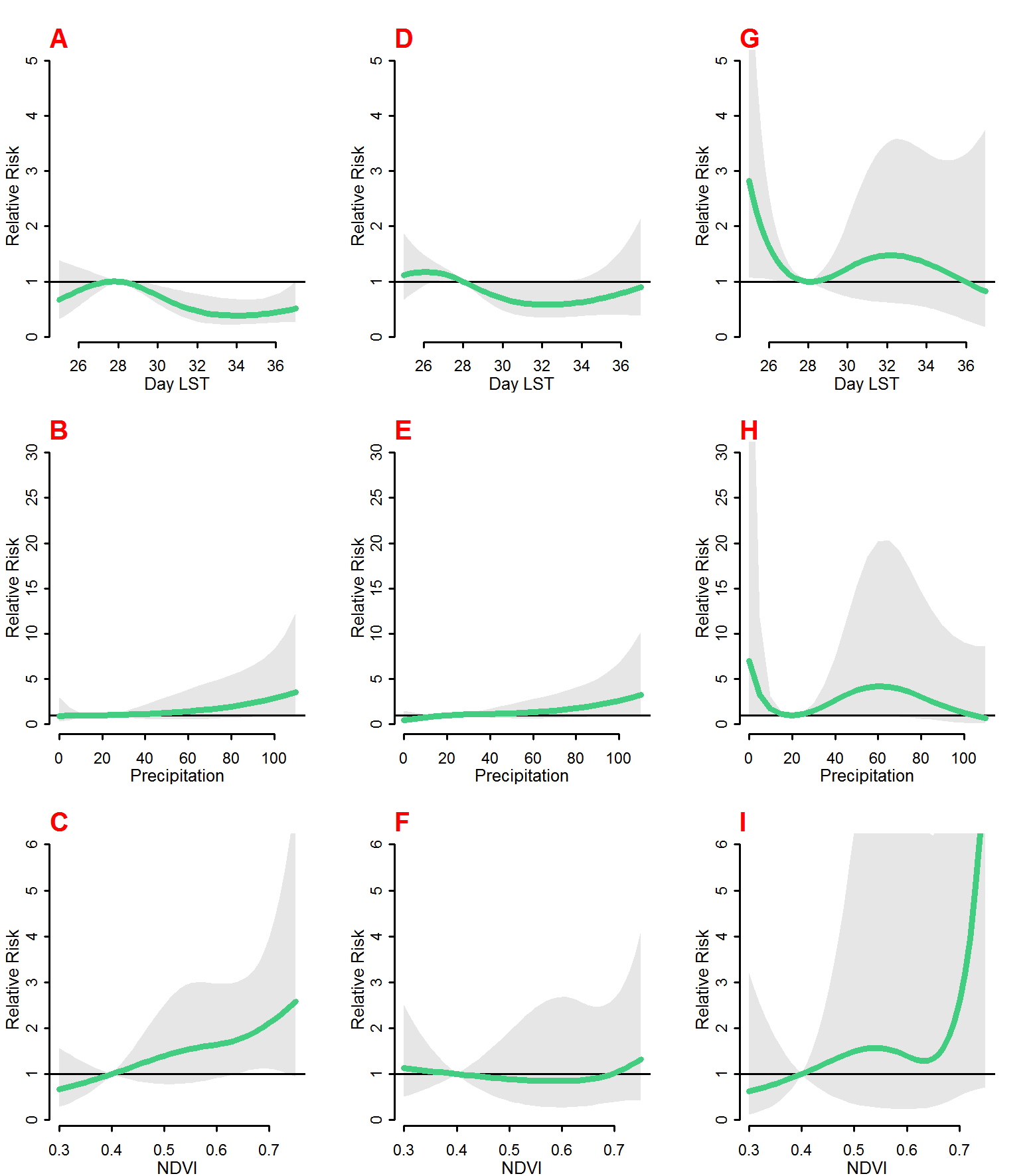

Supplement: S6 Fig — The overall Risk of day LST (°C) (A, D and G), Precipitation (mm) (B, E and H), and NDVI (C, F and I) on malaria mortality in Asembo (A, B and C), Gem (D, E and F) and Karemo (G, H and I) for the whole lag period including smooth of month of year to adjust for seasonality. The bold lines indicate Relative risks while the shaded regions display the 95% Confidence intervals. (TIFF) [file pone.0154204.s007.tiff]
